# Postoperative Sore Throat among Patients Following General Anesthesia with Endotracheal Intubation in a Tertiary Care Centre

**DOI:** 10.31729/jnma.8399

**Published:** 2024-01-31

**Authors:** Sabin Gauchan, Chitra Thapa, Rajiv Yadav, Sabin Bhandari

**Affiliations:** 1 Department of Anesthesiology, Nepal Medical College and Teaching Hospital, Jorpati, Kathmandu, Nepal

**Keywords:** *endotracheal intubation*, *general anaesthesia*, *prevalence*

## Abstract

**Introduction:**

Postoperative sore throat is the second most common minor adverse event after general anaesthesia with endotracheal intubation. It is an uncomfortable and stressful sequel of tracheal intubation. The incidence of postoperative sore throat varies across different studies and type of anesthesia technique used. The aim of the study was to find out the prevalence of postoperative sore throat following general anaesthesia with endotracheal intubation in a tertiary care centre.

**Methods:**

A descriptive cross-sectional study was conducted among the patients who underwent surgery under general anaesthesia with endotracheal intubation from 1 December 2022 to 31 October 2023 after receiving ethical approval from the Institutional Review Committee. The anaesthesia technique was standardized in all the patients. A convenience sampling method was used. The point estimate was calculated at a 95% Confidence Interval.

**Results:**

Among 200 patients, postoperative sore throat was seen in 86 (43%) (36.14-49.86, 95% Confidence Interval) patients. The maximum reported time of sore throat was at a fourth postoperative hour 80 (93.02%).

**Conclusions:**

The prevalence of postoperative sore throat among patients undergoing surgery under general anaesthesia with endotracheal intubation was similar to the studies conducted in similar settings.

## INTRODUCTION

Postoperative sore throat (POST) is the second most common minor adverse event after general anaesthesia.^1^ POST seems to happen only when the airway is manipulated under anaesthesia. Though the aetiology is not yet clearly understood, POST has been associated with mucosal dehydration or oedema, tracheal ischemia secondary to the pressure of endotracheal tube cuffs, aggressive oropharyngeal suctioning, and mucosal erosion from friction between delicate tissues and the endotracheal tube.^2,3^

Most of the measures that have been recommended for reducing POST have been directed at limiting the physical trauma that results from airway instrumentation and manipulation. We adopt every measure, which is possible in our setup, to reduce the occurrence of POST. However, much less is known about the exact magnitude of POST with improved anaesthesia practices.

The aim of the study was to find out the prevalence of postoperative sore throat following general anaesthesia with endotracheal intubation in a tertiary care centre.

## METHODS

This descriptive cross-sectional study was conducted from 1 December 2022 to 31 October 2023 at Nepal Medical College and Teaching Hospital after approval from the Institutional Review Committee (Reference number: 31-079/080). Among the patients who underwent elective surgery under general anaesthesia with endotracheal intubation, those in the age group of 18-65 years with the American Society of Anesthesiologists (ASA) Physical Status Grade I, II and III and provided consent were included. Patients with impaired cognitive ability, those with a history of recent or ongoing upper respiratory tract infection, surgeries within the area of the mouth, pharynx, larynx and throat and use of double lumen tube were excluded from the study. A convenience sampling method was used. The sample size was calculated using the following formula:


$$\begin{array}{ll} \text{n} & ={\text{Z}}^{2}\times \frac{\text{p}\times \text{q}}{{\text{e}}^{\text{2}}}\\ & =1.96^{2}\times \frac{0.488\times 0.512}{0.07^{2}}\\ & =196 \end{array}$$

Where,

n = minimum required sample size
Z = 1.96 at 95% Confidence Interval (CI)
p = prevalence which is taken as 48.8% from the previous study.^4^
q = 1-p
e = margin of error, 7%

The minimum required sample size was 196. However, a total of 200 patients were enrolled in this study. Data collection was done in the post-anesthesia care unit (PACU). The time of admission to the postanesthesia care unit has marked the beginning of the 24-hour observation period. From the anesthesia record patient's demography, ASA physical status and information regarding the type and duration of surgery, patient position during surgery, number of attempts at intubation, and duration of intubation were noted. The presence or absence of POST was questioned by either pain or discomfort or both during swallowing at 2, 4, 6, 12 and 24 hours post-extubation. The sore throat was evaluated using a 0-100 mm visual analogue scale (VAS), with 0= no pain or discomfort to 100= worst pain imaginable. A VAS of 0 was recorded as absence and all other VAS values were recorded as presence of POST. Sore throat if present was also evaluated using the standard rating system presented by Farhang and Grondin. Grade 0= no sore throat, Grade 1= mild sore throat (complaints of sore throat only on asking); Grade 2= moderate sore throat (complaints of sore throat on his/her own); Grade 3= severe sore throat (change of voice or hoarseness, associated with throat pain).^5^

Data was entered and analyzed using IBM SPSS Statistics version 16.0. The point estimate was calculated at a 95% CI.

## RESULTS

Among 200 patients following general anaesthesia with endotracheal intubation, the prevalence of POST was seen in 86 (43%) (36.14-49.86, 95% CI) patients. The average age of the patients with POST was 42.62±10.26 years. Among them 51 (59.30%) were females. The ASA grading was I in 41 (47.67%) and II in 45 (52.33%) patients. A total of 14 (16.38%) patients were overweight. Among 86 patients, 16 (18.60%) were smokers (Table 1).

**Table 1:** Factors related to anaesthesia and surgery (n = 86).

Variables	n (%)
**Type of surgery**	
General	41 (47.67)
Urology	24 (27.91)
Gynaecology	21 (24.41)
**CL grade**	
I	24 (27.9)
II	60 (69.8)
III	2 (2.30)
**Intubation attempts**	
1	53 (61.60)
2	24 (27.90)
3	9 (10.50)
**Duration of the tube in place (min)**	
<60	17 (19.80)
60-120	59 (68.60)
>120	10 (11.60)
**Patient positioning**	
Supine	75 (87.20)
Lithotomy	11 (12.79)

The maximum reported time of sore throat was at a fourth postoperative hour 80 (93.02%) (Table 2).

**Table 2:** Postoperative hours of patients having POST (n= 86).

Postoperative period (hours]	n (%)
Second	68 (79)
Fourth	80 (93.02)
Sixth	33 (38.4)
Twelfth	-
Twenty-fourth	-

On the fourth post-operative period, grade 3 severity was seen among 16 (18.60%) (Table 3).

**Table 3:** Severity of POST at different postoperative hours (n= 86).

Postoperative period (hours)	Severity	n (%)
Second	0	18 (20.93)
	1	44 (51.16)
	2	24 (27.90)
	3	-
Fourth	0	6 (6.97)
	1	40 (46.51)
	2	24 (27.90)
	3	16 (18.60)
Sixth	0	53 (61.62)
	1	28 (32.55)
	2	5 (5.81)
	3	-

## DISCUSSION

Among 200 patients, POST was seen in 86 (43%). A study reported a POST prevalence of 59.6% after tracheal intubation.^6^ In their study, few of the patients required a nasogastric tube intraoperatively. A significant association between nasogastric tube and postoperative sore throat was also reported. Another study has also confirmed a higher POST in intubated patients who required nasogastric tube.^7^ None of the patients required intraoperative nasogastric tube placement in our study which might be the reason for a lower POST prevalence.

In another similar study, a much higher prevalence of 61.8% has been reported.^8^ In their study, the duration of anaesthesia was less than 1 hour in 57.9%, 1 to 2 hours in 27.6% and more than 2 hours in 14.5% of patients. They found the duration of anaesthesia to be an associated factor for POST. In our study, the duration of anaesthesia was less than 1 hour in 68.60%, 1 to 2 hours in 19.80% and more than 2 hours in 11.60% of patients. A longer duration of anaesthesia and the use of an intraoperative nasogastric tube might be the reason for a higher prevalence in their study.

Studies support use of manometers following tracheal intubation to measure and maintain cuff pressure to a minimum for proper seal.^9^ A POST prevalence of 34% has been reported in the group in which endotracheal tube cuff pressure was adjusted using a manometer.^9^ In our institute, we do not have access to a manometer to monitor tracheal tube cuff pressure. We inflate the cuff with a minimum volume of air that prevents leaks during positive pressure ventilation. But tracheal tube cuff pressure estimated by palpation with personal experience is found to be much higher than measured or what may be optimal.^9^

We found that 59.3% of the patients who had sore throats were females. Being female is identified as a risk factor for POST.^10,11^ But in studies that used a 6.0 mm ID (internal diameter) rather than 7.0 mm ID tracheal tubes in female patients, no significant difference was found in males and females.^12^ A much lower POST prevalence has been reported in females who were intubated with 6.0 mm ID tracheal tubes as compared to 7 mm ID tracheal tubes (27.1% vs 51.1%). In our practice, we use 7.0 mm ID tracheal tubes in adult females and 7.5 mm ID tracheal tubes in adult males.^13^ Due to anatomical differences in the larynx between males and females, a 7.0 mm ID tracheal tube may be large and produce a tight fit in females resulting in POST. Use of 6.5 mm ID or 6.0 mm ID tracheal tubes rather than 7.0 mm ID tubes for females may help reduce the occurrence of POST in our population.

In our institute, most of the intubations are performed by Anesthesia residents under the supervision of a consultant anaesthetist. This might be the reason that despite very less patients with a Cormack Lehane grade of III (2.3%) in our study, 33 (38.40%) patients required multiple attempts at intubation. The literature is inconsistent with regard to the association of the expertise of anaesthetists performing intubation and POST.

The maximum reported time of POST was at the 4th postoperative hour (93.02%). The number of patients with POST decreased to 33 (38.4%) at the 6th postoperative hour. By the twelfth postoperative hour, POST was spontaneously resolved in all the patients. None of the patients reported severe POST. Though regarded as minor complications, post-anaesthetic throat complaints rank amongst the most frequent subjective complaints besides nausea and vomiting. POST is distressing for the patient, often remains an unpleasant memory and causes significant patient dissatisfaction. Hence, knowing the incidence and developing a better appreciation of POST is necessary to improve patient satisfaction with anaesthetic care.

An association between various surgical or anaesthetic factors and the occurrence and severity of POST could not be made in this study due to a small sample size and the nature of the study design. Also, the single-centric nature of the study could limit the generalizability of the findings of this study.

## CONCLUSIONS

The prevalence of postoperative sore throat among patients undergoing surgery under general anaesthesia with endotracheal intubation in our practice was similar to the other studies conducted in similar settings. We suggest studies with higher level of evidence for the identification of risk factors and the use of prophylactic measures to reduce the occurance of POST.
